# The clinical and economic benefits of capecitabine and tegafur with uracil in metastatic colorectal cancer

**DOI:** 10.1038/sj.bjc.6603738

**Published:** 2007-04-17

**Authors:** J Almond, S Ralston

**Affiliations:** 1 Merck Pharmaceuticals, Harrier House, West Drayton, Middlesex UB7 7QG, UK

**Sir**,

With reference to the study by Ward *et al* (2006), the article compares the cost effectiveness of oral fluoropyrimidine treatment for mCRC *vs* intravenously administered therapies. In particular, this paper compares Uftoral® (tegafur–uracil), with capecitabine, based on unit costs for drugs as outlined in the British National Formulary, 2002. However, there are a number of discrepancies we would like to highlight with this assessment. First, as of March 2006 the price of Uftoral® (tegafur–uracil) was reduced as indicated in the following table:


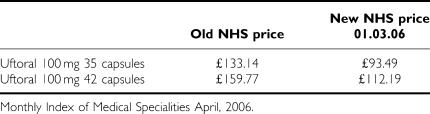


Given this significant change in price, over a standard three cycles of treatment (105 days), the new price represents a 59% reduction in drug costs (including costs of folinic acid). If the cost minimisation assessment as completed by Ward *et al* is replicated and we include the new NHS price for Uftoral®, the treatment costs for a 12-week course of tegafur with uracil would be £1788. This is lower than any of the other therapies referred to in the article and represents a cost saving *vs* capecitabine of £343.1 over a 12-week period.

We believe that there are also inaccuracies in the estimation of costs related to adverse events included within the model. In table 5, Ward *et al* report the frequency of consultations and hospitalisations relating to adverse events over the treatment period for capecitabine, UFT/LV (Uftoral®) and the Mayo regimens. The sources referenced for UFT/LV are Roche (2002) and Pazdur *et al* (1999). In assessing Pazdur *et al* (1999), information as included in table 5 of Ward *et al*, is not reported in this publication. Additionally, we believe the reference, ‘Roche (2002) *‘*Xeloda (capecitabine): achieving clinical excellence in the treatment of metastatic colorectal cancer. Roche submission to the National Institute of Clinical Excellence’, to be either incorrect or inappropriate as this source cannot be referenced. Therefore, we question whether the stated figures for UFT/LV for, ‘A and E’, ‘Clinic Consultation’ and ‘Other hospital visits’ are appropriate for inclusion in such an analysis. We also dispute why these categories would have a zero frequency of incidence for patients treated with capecitabine, yet be an issue with UFT/LV.

Finally, we would also dispute how the adverse event costs for Uftoral® are assumed to be equal to that of the Mayo regimen. We believe that this is an overestimation and biased against Uftoral®. We would propose that adverse event costs for Uftoral® are more likely to be lower or at least equal to capecitabine, given the incidence of hand and foot syndrome as a prominent adverse event for capecitabine.

Given the points raised, the article by Ward *et al*, misrepresents the current costs of treating metastatic colorectal cancer with Uftoral® and this could result in clinicians being less willing to include Uftoral® in their treatment protocols.
